# Kratom Consumption Associated With Herb-Induced Liver Injury and a Pharmacokinetic Interaction: A Case Report

**DOI:** 10.7759/cureus.106016

**Published:** 2026-03-28

**Authors:** Samantha Gnanasegaram, Corneliu N Stanciu

**Affiliations:** 1 Psychiatry, Hope Counseling Services, Pittsfield, USA; 2 Psychiatry, Dartmouth Hitchcock Medical Center, Concord, USA

**Keywords:** cyp-mediated interactions, hepatotoxicity, herbal supplements, herb-induced liver injury, kratom

## Abstract

Kratom and kratom products are increasingly used in Western countries for pain management, mood disorders, and opioid withdrawal. Despite perceptions of safety, kratom can cause hepatotoxicity and clinically relevant herb-drug interactions, particularly with medications metabolized by cytochrome P450 (CYP) enzymes.

We report a 45-year-old male with stable psychiatric conditions on nortriptyline and lisdexamfetamine, who developed mild, reversible liver enzyme elevations and supratherapeutic nortriptyline levels during chronic kratom use (raw, dry leaf, two tablespoons daily for five to six years). Initial labs showed aspartate aminotransferase (AST) 33 U/L, alanine aminotransferase (ALT) 70 U/L, alkaline phosphatase (ALP) 136 U/L, gamma-glutamyl transferase (GGT) 141 U/L, and nortriptyline 350 mcg/L. Dose reduction of nortriptyline partially corrected serum levels, but liver enzyme elevations persisted. Kratom discontinuation led to normalization of both liver function tests and nortriptyline levels within three weeks.

This case illustrates two clinically important considerations: kratom-induced liver injury (KILI) and a CYP-mediated herb-drug interaction. The patient’s use of raw leaf contrasts with commercial products, which may include concentrated extracts, 7-hydroxymitragynine (7OHMG)-enriched formulations, or ethanol-based preparations, potentially increasing hepatotoxicity and interaction risk.

Even mild liver enzyme elevations in patients using kratom warrant evaluation, particularly with concurrent CYP-metabolized medications. Clinicians should educate patients about product variability, monitor liver function and drug levels, and consider temporary kratom discontinuation to assess causality. This case reinforces the growing evidence of kratom’s hepatotoxic potential and the importance of integrating pharmacokinetic considerations into patient care.

## Introduction

Kratom (*Mitragyna speciosa*) is a tropical tree native to Southeast Asia, whose leaves have traditionally been used ethnomedically in its indigenous habitat for centuries [1]. Over the past decade, kratom has gained popularity in Western countries for self-management of pain, mood disorders, and opioid withdrawal, among other indications [2]. This trend is particularly notable among clinical populations, who often receive conventional medical treatments and perceive kratom as a “natural” and relatively safe substance [3,4]. Emerging evidence demonstrates that kratom, especially commercial products labeled as “kratom,” can have clinically significant adverse effects, including hepatotoxicity and herb-drug interactions [5,6].

The psychoactive effects of kratom are primarily attributed to its indole alkaloids, with mitragynine (MG) being the most abundant and 7-hydroxymitragynine (7OHMG) the most pharmacologically potent. MG acts as a partial agonist at the μ-opioid receptor, with additional activity at δ- and κ-opioid receptors, though with lower affinity. It also exhibits non-opioid receptor activity, including antagonism at 5-HT2A receptors and agonism at α2-adrenergic receptors, which may contribute to its analgesic and stimulant-like effects at lower doses. 7OHMG, a minor metabolite, demonstrates higher affinity and efficacy at the μ-opioid receptor, producing opioid-like analgesia and reinforcing effects.

Unlike classical opioids, MG and related alkaloids appear to function as G-protein biased agonists at the μ-opioid receptor, with relatively limited recruitment of β-arrestin pathways. This signaling profile has been hypothesized to confer a lower risk of respiratory depression compared to traditional opioids, although clinically significant toxicity and fatalities have still been reported - particularly in the context of polysubstance use and when commercial products are involved. Additionally, kratom alkaloids interact with cytochrome P450 (CYP) enzymes (notably CYP3A4, CYP2D6, and CYP2C9), creating the potential for pharmacokinetic drug interactions.

Drug-induced liver injury (DILI) is a leading cause of acute liver failure (ALF) in the United States, with clinical presentations ranging from asymptomatic enzyme elevations to severe liver dysfunction. [7] While DILI is often defined by alanine aminotransferase (ALT) or aspartate aminotransferase (AST) levels exceeding five times the upper limit of normal (ULN), alkaline phosphatase (ALP) elevations greater than two times the ULN, or bilirubin elevations over two times the ULN with associated transaminase increases [8], milder or subclinical elevations are increasingly recognized - particularly with herbal and dietary supplements. The incidence of liver injury attributed to these supplements has been rising, underscoring the need to identify cases even when enzyme elevations are modest [9].

In this report, we present a case of a middle-aged patient with stable psychiatric conditions who developed mild, reversible liver enzyme elevations and elevated nortriptyline levels during chronic kratom use. Notably, the normalization of both liver enzymes and nortriptyline levels following kratom discontinuation suggests not only herb-induced liver injury (HILI) but also a clinically relevant herb-drug interaction affecting the metabolism of nortriptyline. This case emphasizes the importance of monitoring for hepatotoxicity and altered pharmacokinetics in patients using kratom concurrently with prescription medications.

## Case presentation

A 45-year-old, engaged, Caucasian male with no prior family medical or psychiatric history presented to an outpatient psychiatric clinic for medication management. His psychiatric history was notable for major depressive disorder, generalized anxiety disorder, and attention-deficit/hyperactivity disorder, inattentive type. He had no history of psychiatric hospitalizations or trauma. He was a computer systems analyst and had been psychiatrically stable for several years on nortriptyline (Pamelor) 150 mg nightly and lisdexamfetamine (Vyvanse) 30 mg daily, with no recent dose changes. 

The patient reported chronic low back pain related to a remote injury, approximately 15 years prior, sustained after a fall from a rock-climbing wall, resulting in vertebral compression fractures. No recent radiologic evaluations had been performed, though imaging had been obtained at the time of injury. To manage this pain, he independently initiated kratom use approximately five to six years prior, without recommendation from a healthcare provider. He used a commercially available “wildcraft” product in the form of crushed dried leaf powder, typically consuming approximately two tablespoons daily in divided doses (once to twice daily), a regimen he arrived at through trial and error, based on perceived efficacy.

He reported intermittent breaks from kratom use, typically during vacations, occurring approximately twice per year and lasting five to seven days. He denied experiencing withdrawal symptoms during these periods. He also denied cravings, dose escalation, loss of control, or other features suggestive of a substance use disorder. He did not consume alcohol or tobacco and reported occasional cannabis use, typically smoking once nightly to aid with sleep.

At the initial evaluation in April 2025, while taking nortriptyline 150 mg nightly and continuing kratom, laboratory testing revealed mildly elevated liver enzymes: AST 33 U/L, ALT 70 U/L, ALP 136 U/L, total bilirubin 0.7 mg/dL, and gamma-glutamyl transferase (GGT) 141 U/L. Nortriptyline levels were elevated at 350 mcg/L. Urine toxicology was positive for amphetamine (1050 ng/mL), tetrahydrocannabinol (THC) (725 ng/mL), and MG (447 ng/mL). A mental status examination was performed as part of routine psychiatric care and was unremarkable. No formal psychometric assessments were administered. A physical or neurological examination was not conducted at that visit. The patient was clinically asymptomatic.

Repeat laboratory testing in October 2025 demonstrated persistent mild abnormalities: AST 27 U/L, ALT 55 U/L, ALP 92 U/L, total bilirubin 0.4 mg/dL, GGT 162 U/L, and nortriptyline 323 mcg/L. Urine toxicology remained positive for MG (167 ng/mL). The lisdexamfetamine dose remained unchanged throughout this period.

Given the persistently elevated nortriptyline levels, the Pamelor dose was reduced to 75 mg nightly in mid-November 2025, while kratom use remained unchanged. Laboratory monitoring three weeks later (November 17, 2025) showed AST 33 U/L, ALT 62 U/L, ALP 70 U/L, total bilirubin 0.4 mg/dL, and GGT 137 U/L. Nortriptyline levels decreased to 132 mcg/L. Urine toxicology revealed amphetamine 2848 ng/mL, THC 382 ng/mL, and MG 1193 ng/mL. Despite the dose reduction, mild hepatic enzyme elevations persisted.

The patient was subsequently educated regarding the potential hepatotoxic effects of kratom and possible herb-drug interactions. He agreed to abruptly discontinue kratom while continuing nortriptyline 75 mg nightly and lisdexamfetamine 30 mg daily. Following discontinuation, his chronic back pain was managed with a combination of ibuprofen and acetaminophen as needed, which resulted in partial relief - he was not pain-free but remained functionally able to perform daily activities.

At follow-up approximately three weeks later (December 27, 2025), laboratory studies demonstrated normalization of liver function tests: AST 19 U/L, ALT 32 U/L, ALP 68 U/L, total bilirubin 0.5 mg/dL, and GGT 22 U/L. Nortriptyline levels decreased further to 58 mcg/L. Urine toxicology was negative for MG, though it remained positive for amphetamine (1431 ng/mL) and THC (224 ng/mL). The patient remained clinically stable, with no evidence of hepatotoxicity, psychiatric decompensation, or withdrawal symptoms. Nortriptyline was continued at 75 mg nightly at the time of the last follow-up.

Throughout the observation period, additional laboratory evaluations, including the complete metabolic panel, complete blood count, thyroid-stimulating hormone, and hepatitis panel, all remained within normal limits. Laboratory trends are summarized in Table 1.

**Table 1:** Laboratory Results Over Time in a Patient Using Kratom While on Nortriptyline. ↑ indicates values above the upper limit of normal. Reference ranges are provided in column headers. AST: aspartate aminotransferase; ALT: alanine aminotransferase; ALP: alkaline phosphatase; GGT: gamma-glutamyl transferase

Date	AST (10-40 U/L)	ALT (9-46 U/L)	ALP (36-130 U/L)	Total Bilirubin (0.2-1.2 mg/dL)	GGT (3-95 U/L)	Nortriptyline (50-150 mcg/L)	Key Events
April 2025	33	70 ↑	136 ↑	0.7	141 ↑	350 ↑	Baseline on Pamelor 150 mg + kratom
October 2025	27	55 ↑	92	0.4	162 ↑	323 ↑	Continued kratom use
November 17, 2025	33	62 ↑	70	0.4	137 ↑	132	Pamelor reduced to 75 mg
December 27, 2025	19	32	68	0.5	22	58	Kratom discontinued ×3 weeks

## Discussion

This case illustrates two clinically important considerations: kratom-associated hepatotoxicity and a potential herb-drug interaction with nortriptyline, both supported by temporal associations and biochemical trends observed in this patient.

Kratom-induced liver injury (KILI)

KILI, a subset of HILI, is increasingly recognized in the literature. The pathophysiology of kratom-associated hepatotoxicity is not fully elucidated but is likely multifactorial. Proposed mechanisms include direct hepatocellular toxicity from kratom alkaloids, mitochondrial dysfunction, oxidative stress, and immune-mediated idiosyncratic reactions. In vitro studies suggest that MG and its active metabolite, 7OHMG, may impair hepatic bile transporters and disrupt mitochondrial respiration, contributing to cholestatic or mixed-pattern liver injury. Additionally, inhibition of CYP enzymes may lead to the accumulation of hepatotoxic metabolites or interacting drugs, further increasing hepatic burden. 7OHMG, the minor yet highly potent indole alkaloid, mainly formed via hepatic metabolism of MG, may also contribute disproportionately to the toxic effects of kratom products enriched through processing or extraction. 

Our patient demonstrated mild, reversible liver enzyme elevations with a predominantly cholestatic pattern, with an R factor (ratio of ALT to ALP, normalized to their upper limits of normal) of ~1.5-2.1, consistent with prior reports [10,11]. While his transaminase elevations were modest and did not reach classical DILI thresholds (ALT ≥ 5× ULN and ALP ≥ 2× ULN), the temporal relationship with kratom use and normalization upon discontinuation support a diagnosis of HILI.

Causality can be assessed using the Roussel Uclaf Causality Assessment Method (RUCAM), a structured and validated scoring system used to evaluate the likelihood that a substance caused liver injury [12]. RUCAM incorporates factors such as time to onset, course after discontinuation, risk factors, concomitant medications, exclusion of alternative causes, and prior evidence of hepatotoxicity. Scores categorize causality as excluded, unlikely, possible, probable, or highly probable. In this case, the RUCAM score would likely fall within the “probable” range.

Systematic reviews and case reports corroborate this pattern. Schimmel and Dart [13] reviewed reported human cases and found that KILI often occurs within two to three weeks of use, presents with cholestatic or mixed patterns, and is generally reversible after discontinuation. Roma et al. [14] identified 69 cases, noting that 80% exhibited cholestatic liver injury, with a subset requiring hospitalization or liver transplantation. Most cases resolved upon kratom discontinuation, though rare severe outcomes occurred. In our patient, liver function normalized without supportive care, aligning with the milder spectrum reported in the literature. These findings reinforce that kratom can cause subclinical or mild HILI, which can be overlooked unless specifically monitored, and highlight the broad range of clinical presentations, ranging from asymptomatic enzyme elevations to fulminant liver failure [13,14].

Importantly, this patient consumed raw, dry-leaf kratom. Available products diverge from the natural leaf widely in composition: some are enriched in 7OHMG, others are concentrated extracts, and preparation methods (e.g., ethanol or other solvent extractions) can alter alkaloid content [15]. These variations may increase systemic exposure to hepatotoxic or CYP-inhibiting alkaloids. Systematic review data suggest that concentrated or adulterated preparations may pose a higher risk for liver injury and herb-drug interactions than raw-leaf products, highlighting the need for careful product assessment and patient counseling [16].

Herb-drug interaction with nortriptyline

Nortriptyline is predominantly metabolized by CYP2D6, with minor contributions from CYP1A2 and CYP2C19. Kratom alkaloids, particularly MG, are metabolized via CYP3A4 and CYP2D6, and have been shown in vitro to inhibit these enzymes [17,18]. In this patient, supratherapeutic nortriptyline levels (350 mcg/L, reference 50-150 mcg/L) were observed during concurrent kratom use. After a 50% dose reduction and kratom discontinuation, levels decreased to 132 and 58 mcg/L, respectively. This temporal pattern strongly suggests a clinically relevant CYP-mediated herb-drug interaction, whereby kratom inhibited nortriptyline metabolism, leading to its accumulation. Although the patient remained asymptomatic, this interaction has important clinical implications, particularly for medications with narrow therapeutic indices. Elevated tricyclic antidepressant levels can increase the risk of anticholinergic toxicity, arrhythmias, and central nervous system effects. Variability in kratom product composition may further amplify these risks.

Pharmacokinetic considerations

Controlled human studies demonstrate that MG has a prolonged elimination half-life (~40-68 hours) and reaches steady state after seven to nine days of repeated dosing [19]. This sustained systemic exposure, coupled with enzyme inhibition, explains why chronic daily use, even at moderate doses, can lead to hepatotoxicity and herb-drug interactions over time. The systematic review also notes that the majority of hepatotoxic cases occurred with repeated use over days to weeks, supporting the temporal- and dose-dependent nature of these effects. Variability in product concentration or extraction method may further increase systemic exposure and risk.

Adverse effects, contraindications, and precautions

Kratom use has been associated with a wide range of adverse effects. Common effects include nausea, vomiting, constipation, dizziness, sedation, and pruritus. At higher doses, and depending on whether commercially available products are involved, users may experience seizures, QTc prolongation, psychosis, and even respiratory depression. Chronic use has been linked to dependence, withdrawal symptoms, endocrine disturbances, and weight loss. Hepatotoxicity, as demonstrated in this case, is an increasingly recognized complication [20,21].

Contraindications and precautions include use in patients with pre-existing liver disease, those taking medications metabolized by CYP450 enzymes (particularly CYP2D6 and CYP3A4 substrates), and individuals with psychiatric and substance use disorders. Caution is also warranted in patients taking serotonergic agents, due to potential additive effects and risk of serotonin syndrome. Additionally, kratom products may be adulterated with other substances, including opioids or synthetic compounds, further increasing risk.

Clinical implications

This case underscores several important considerations for clinical practice. Even mild elevations in liver enzymes among patients using kratom should prompt further evaluation and ongoing monitoring, particularly during the initial weeks of use, when the risk of hepatotoxicity appears to be highest. A thorough medication review is also essential, with particular attention to concomitant agents metabolized through CYP pathways (e.g., CYP2D6 and CYP3A4), given the potential for clinically significant pharmacokinetic interactions.

Equally important is patient education. Individuals should be counseled that “natural” products, including kratom and commercially labeled “kratom” products, are not inherently safe. Substantial variability in product composition and alkaloid content may increase the risk of hepatotoxicity and clinically significant drug-drug interactions. From a harm reduction perspective, temporary discontinuation of kratom can be a useful strategy to clarify causality and mitigate risk, while avoiding unnecessary or abrupt treatment changes when clinically appropriate. These considerations also highlight the need for improved regulatory oversight, including standardized manufacturing practices, accurate labeling of alkaloid content, and clearer dosing guidelines to reduce variability and prevent toxicity.

In line with CARE (CAse REport) guidelines, the patient was followed longitudinally [22]. At the most recent follow-up, approximately three weeks after kratom discontinuation, liver function tests had normalized (AST 19 U/L, ALT 32 U/L, ALP 68 U/L, GGT 22 U/L), and nortriptyline levels had returned to the therapeutic range (58 mcg/L). Although his chronic low back pain persisted, it remained manageable with over-the-counter ibuprofen and acetaminophen, allowing him to maintain functional daily activity. These observations further emphasize the importance of considering safer, evidence-based alternatives for pain management, including behavioral interventions, adjuvant pharmacologic therapies, and referral to a pain management specialist when discontinuing kratom.

Strengths

A key strength of this case is the longitudinal monitoring of both liver function tests and nortriptyline serum levels, allowing for a clear temporal association with kratom use and discontinuation. The availability of objective toxicology data, including MG levels, further strengthens the causal inference. Additionally, the patient’s relative clinical stability and absence of confounding comorbidities enhance the interpretability.

Limitations

This is a single case report; confounding factors (e.g., cannabis use, undetected hepatotoxins) cannot be fully excluded. While the RUCAM score suggests probable HILI, a rechallenge was not performed for safety reasons. Direct causality between kratom and elevated nortriptyline levels remains inferential, based on temporal association and known CYP-mediated interactions. The systematic review further emphasizes that most literature consists of case reports, highlighting the need for prospective studies to clarify incidence, risk factors, and dose-response relationships.

Future directions

Future research should focus on prospective studies to better define the incidence, dose-response relationships, and risk factors for kratom-associated hepatotoxicity. Standardization of kratom products, including quantification of alkaloid content such as MG and 7OHMG, is critical. Further investigation into CYP-mediated interactions, particularly in patients on psychotropic medications, is warranted. Mechanistic studies exploring mitochondrial toxicity, bile transporter inhibition, and genetic susceptibility may help clarify pathophysiology. Finally, the development of clinical guidelines for screening, monitoring, and counseling patients using kratom would improve safety in clinical practice.

## Conclusions

Chronic kratom use may lead to mild, reversible cholestatic liver injury and can result in clinically significant interactions with CYP-metabolized medications, such as nortriptyline. Even modest liver enzyme elevations should prompt assessment for herbal supplement use, particularly in patients on prescription medications. Product variability - including raw leaf versus concentrated or extracted forms - may further increase hepatotoxic and pharmacokinetic risk. Clinicians should maintain vigilance for both hepatotoxicity and herb-drug interactions, educate patients regarding potential risks, and consider monitoring liver function and drug levels when kratom use is ongoing. This case adds to the growing body of evidence from systematic reviews and case reports, demonstrating that kratom carries hepatotoxic potential and underscores the importance of integrating pharmacokinetic considerations into patient care.
